# Risk factors associated with intraoperative shivering during caesarean section: a prospective nested case-control study

**DOI:** 10.1186/s12871-022-01596-7

**Published:** 2022-02-28

**Authors:** Xiaofei Qi, Daili Chen, Gehui Li, Jun Cao, Yuting Yan, Zhenzhen Li, Feilong Qiu, Xiaolei Huang, Yuantao Li

**Affiliations:** Department of Anesthesiology, Shenzhen Maternity & Child Healthcare Hospital, The First School of Clinical Medicine, Southern Medical University, No.2004 Hongli Road, Shenzhen, 518028 China

**Keywords:** Pregnancy, Cesarean section, Shivering, Anxiety, Emergency surgery

## Abstract

**Background:**

To study the incidence and risk factors of shivering in pregnant women during cesarean section.

**Methods:**

We performed a prospective nested case-control study involving parturients scheduled for cesarean sections between July 2018 and May 2021. The overall incidence of intraoperative shivering and its potential risk factors were investigated. The potential risk factors evaluated were pain, anxiety, emergency surgery, transfer from the delivery room, epidural labor analgesia, membrane rupture, labor, and the timing of the surgery. Shivering and body temperature at different time points during the cesarean section were also recorded. The selected seven time points were: entering the operating room, post-anesthesia, post-disinfection, post-delivery, post-oxytocin, post additional hysterotonics, and before leaving the operating room.

**Results:**

We analyzed 212 cesarean section parturients. The overall incidence of shivering was 89 (42.0%). Multivariate logistic regression showed that anxiety, emergency delivery, and transfer from the delivery room to the operating room increased the overall shivering incidence (odds ratio = 1.77, 2.90, and 3.83, respectively). The peak shivering incidence occurred after skin disinfection (63, 29.7%), and the lowest body temperature occurred after oxytocin treatment (36.24 ± 0.30 °C). Stratified analysis of surgery origin showed that emergency delivery was a risk factor for shivering (odds ratio = 2.99) in women transferred from the obstetric ward to the operating room.

**Conclusion:**

Shivering occurred frequently during cesarean sections, with the peak incidence occurring after skin disinfection. Anxiety, emergency delivery, and transfer from the delivery room to the operating room increased the risk of shivering development during cesarean sections.

**Trial registration:**

The study protocol was registered online at China Clinical Registration Center (registration number: ChiCTR-ROC-17010532, Registered on 29 January 2017).

## Background

Shivering involves involuntary rapid skeletal muscle contractions and body tremors and is a common problem during surgical operations [1, 2]. It occurs more frequently during cesarean sections, with a reported incidence between 10 and 85% [3–8], probably due to altered physiology during pregnancy. In addition to causing an unpleasant feeling and interfering with clinical monitoring [9–11], shivering can also increase oxygen consumption and may be associated with a higher risk of surgical site bleeding, cardiac events, wound infections, etc. [10, 12]. All of these may result in adverse outcomes for the mother and fetus.

The etiology of shivering is poorly understood.^1^ The treatment approach for shivering is also not defined [13, 14]. The wide variations in reported shivering incidences during cesarean sections suggest that shivering could be influenced by multiple factors. Identifying and minimizing the risk factors associated with intraoperative shivering could reduce the incidence of shivering and improve maternal outcomes. Several previous studies have suggested that anxiety, hypotension, and hypothermia may be associated with intraoperative shivering during cesarean sections [3, 15], but another study failed to demonstrate a relationship between anxiety and shivering during surgical operations [16]. Studies have also suggested that epidural anesthesia might affect shivering, but the results are controversial [17, 18]. In addition, we suspected that membrane rupture or baby delivery might increase shivering because the loss of warm amniotic fluid can decrease the body temperature. Therefore, we explored whether premature membrane rupture would increase the risk of intraoperative shivering. In clinical practice, we observed that pregnant women who undergo emergency delivery or cesarean sections at night seemed to shiver more frequently; hence we studied the influence of the surgery time (daytime versus nighttime) and types of cesarean section (elective versus emergency) on the occurrence of shivering. Other factors, such as patient transfer origin (from delivery room or from obstetric ward) and surgery indications might also affect shivering. These factors have never been studied previously.

We performed a prospective nested case-control study and the aim was to investigate the incidence and risk factors of shivering in pregnant women during cesarean sections.

## Methods

### Study design and participants

We performed a prospective nested case-control study at Shenzhen Maternity and Child Healthcare Hospital, Southern Medical University, China between July 2018 and May 2021. The study protocol was approved by the ethics committee of Shenzhen Maternity & Child Healthcare Hospital and registered online at China Clinical Registration Center (registration number: ChiCTR-ROC-17010532, Registered on 29 January 2017), all methods were carried out in accordance with relevant guidelines and regulations. Written informed consent was obtained from every study participant.

The inclusion criteria were pregnant women who 1) were scheduled for cesarean sections; 2), received intraspinal anesthesia; 3), American Society of Anesthesiologists (ASA) physical status classification of I-II. The exclusion criteria were 1) blood loss > 500 ml during the delivery; 2), anesthesia level lower than T8; 3), switched from intraspinal anesthesia to general anesthesia.

### Anesthesia

The temperature of the operating room was controlled at 23 °C. Once in the operating room, parturients were routinely monitored with an electrocardiogram, non-invasive blood pressure monitor, and pulse oximeter. A peripheral venous access was secured. Parturients received 12 ~ 15 ml/kg intravenous warmed (37 °C) Lactated Ringer’s solution. All intravenous fluids and medications were kept warm during surgery.

Pregnant women were transferred from either the delivery room or the obstetric ward to the operating room for cesarean section. Some parturients transferred from the delivery room received epidural labor analgesia before the transfer. Epidural anesthesia was performed for the parturients transferred post epidural labor analgesia, and spinal anesthesia was performed for those transferred without epidural labor analgesia.

Labor analgesia: Epidural labor analgesia was performed in the delivery room according to the parturient’s request. An epidural catheter was placed at the L2–3 intervertebral epidural space and 0.08% ropivacaine and 0.4 μg/ml sufentanil were infused with a patient-controlled epidural analgesia pump.

Epidural anesthesia: For cesarean section parturients who had received labor analgesia, epidural anesthesia was performed with epidural 2% lidocaine 5 ml + 0.75% ropivacaine 10 ml through the pre-existing epidural catheter. The anesthesia level was measured with an icy metal ball, and epidural morphine 2 mg was administered before the end of surgery.

Spinal anesthesia: For cesarean section parturients without labor analgesia and those from the obstetric ward, spinal anesthesia was performed. The patient was placed in the right chest knee position, and a 27G spinal needle was inserted at the L_3–4_ intervertebral space. After confirming the needle was in the subarachnoid space based on the outflow of cerebrospinal fluid, 0.5% ropivacaine 15 mg, and morphine 0.1 mg were administered at 0.1 ml/s. The anesthesia level was measured with an icy metal ball and controlled between T4 ~ T6. If the anesthesia level was less than T8, the case was excluded from the study. Phenylephrine was given immediately after the spinal anesthesia with its infusion rate adjusted to maintain the blood pressure when necessary. Phenylephrine was administered at 1 μg/kg.min initially and was adjusted according to the blood pressure using an intravenous pump. The blood pressure was maintained within ±20% of the baseline value.

After the intraspinal anesthesia, the patient was placed in the left tilted position or right hip elevated to avoid supine hypotension syndrome. The patient was placed in the supine position when the surgery began. After childbirth, 10 units of intravenous oxytocin and 10 units of intrauterine oxytocin were administered. Additional hysterotonics (carbetocin or hemabate) was given if necessary.

### Monitoring

Shivering and body temperature was recorded at seven time points, specifically the time of operation room entry, post-anesthesia, post-disinfection, post-delivery, post-oxytocin, post additional hysterotonics, and before leaving the operation room.

### Outcomes and potential risk factor measurements

The primary outcome was the overall incidence of shivering and its potentially associated risk factors during cesarean sections. The overall incidence of shivering was defined as the proportion of women who had shivering from the time they entered the operating room to the time they left the operating room. The secondary outcomes were the incidences of shivering with its associated risk factors at different time points and body temperature changes and their relationship with shivering throughout the cesarean section.

Risk factors studied included age, body mass index, gestational age, emergency or elective cesarean section, transfer origin (from the delivery room versus the obstetric ward to the operating room), membrane rupture, time of membrane rupture, stage of labor, labor analgesia, time of analgesia, anxiety score, visual analogue scale (VAS), operation indications, operation time, anesthesia type (spinal anesthesia or epidural anesthesia), anesthesia level, blood loss, and surgery duration.

The degree of shivering was determined with a score from 0 to 3 on the Bedside Shivering Assessment Scale (0, no shivering; 1, mild, localized to neck/thorax; 2, moderate, intermittent involvement of upper extremities/+thorax; 3, severe, generalized shivering or sustained upper extremities shivering), as described previously [19]. VAS was measured at the time of operating room entry with a score of 0 to 10 (0 indicated no pain and 10 indicated the worst pain). The body temperature was measured with an ear thermometer. Anxiety level was determined with a score of 0 to 2 (0, no anxiety; 1, mild anxiety; 2, serious anxiety). The anesthesia sensory level was tested with an icy metal ball, and a sensory level between T4 and T6 was considered indicative of adequate anesthesia. Active labor was defined as regular uterine contractions and cervical dilation of at least 3 cm [20].

### Statistical analysis

Sample size calculation was conducted in PASS software (version 15.0, NCSS, USA). Based on a previously reported incidence of shivering of approximately 35% [4] during cesarean sections with a 95% confidence level, 14% confidence interval width (two sided), and Z of 1.96, the calculated sample size was 191. Considering about 10% drop-out rate, we aimed to collect data on 213 pregnant women in the present study.

Statistical analyses were performed in SPSS (version 22.0, IBM, USA). Continuous data were presented as mean ± standard deviation or median with inter-quartile range, and compared with the independent Student *t* test or Mann-Whitney U test depending on the results of the normality test. Categorical data were presented as proportions and compared using the Pearson Chi-square test or the Fisher exact test as appropriate.

### Risk factor analysis

Bivariate associations between shivering and different risk factors were analyzed with the Student *t* test, Wilcoxon test, Chi-square test, or univariate logistic regression analysis as appropriate. Factors with *P* < 0.2 or clinical significance were included in the multivariate logistic regression analysis. Stepwise forward selection was used to create the final model to evaluate the association between shivering and different risk factors.

## Results

### Baseline characteristics of study participants

**Fig. 1:**
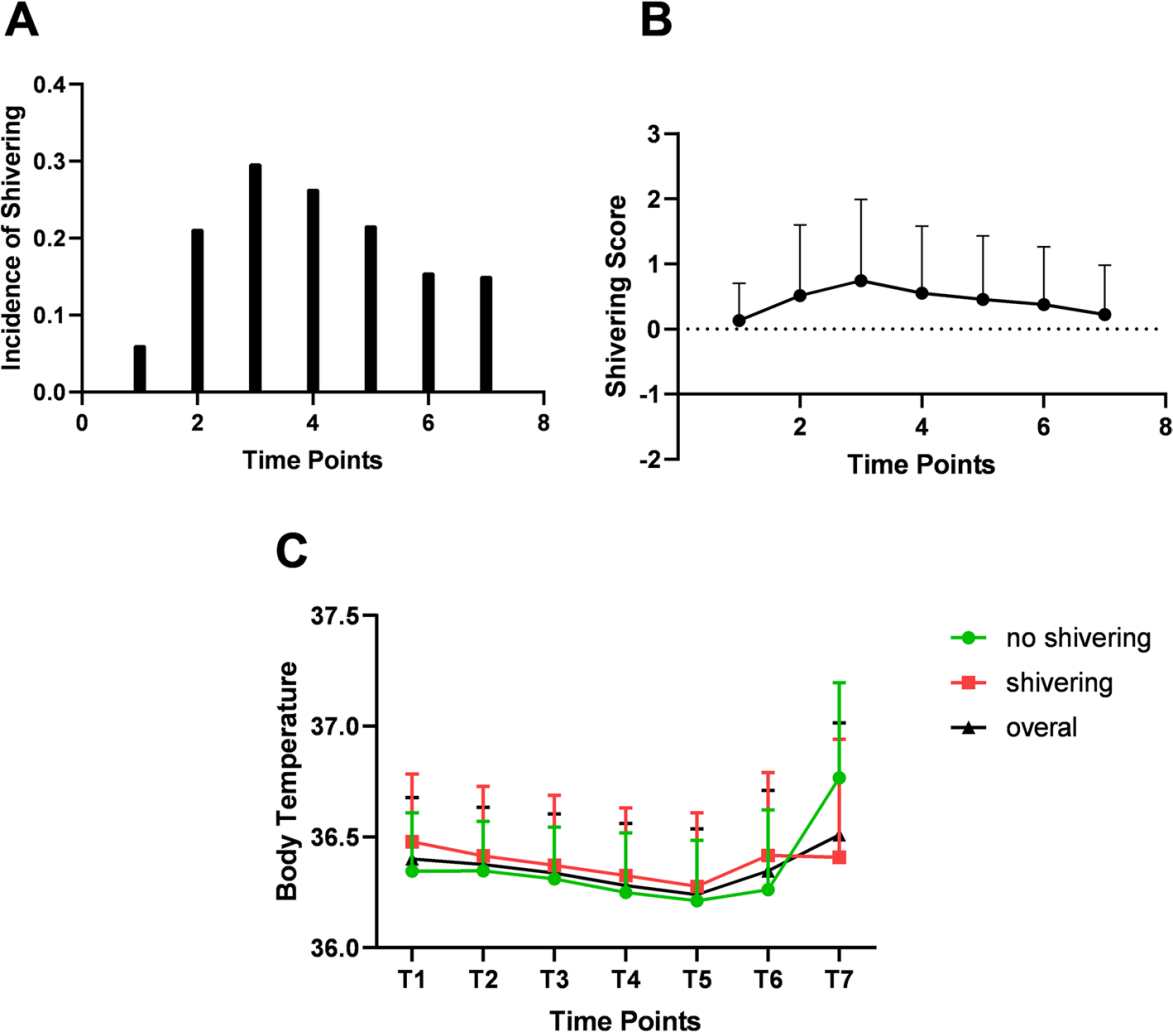
**A**, Incidence of shivering at different time points. The seven time points are the time of entry into the operation room (T1), post-inthrathecal anesthesia (T2), post-abdominal disinfection (T3), post-baby delivery (T4), post-oxytocin administration (T5), post additional hysterotonics (T6), and before leaving the operation room (T7). **B**, Degree of shivering at different time points. **C**. Changes in body temperature at different time points in pregnant women with or without shivering

We evaluated 213 pregnant women and excluded a parturient with intraoperative bleeding > 500 ml. A total of 212 pregnant women were included in the present study. There were 89 pregnant women with shivering during the intraoperative period (overall incidence 42.0%). Comparisons between the pregnant women with and without shivering are listed in Table 1. Age, time of operating room entry, anxiety, VAS, type of delivery, transfer location, analgesia, time of analgesia, membrane rupture, time of membrane rupture, cervical dilation, labor stage, time of labor entry, and operation indications all had statistically significant differences between the two groups. The numbers (proportion) of pregnant women with shivering at different intraoperative time points were 13 (6.1%), 45 (21.2%), 63 (29.7%), 56 (26.4%), 46 (21.7%), 33 (15.6%), and 32 (15.1%) when entering the operating room, post-anesthesia, post-disinfection, post-delivery, post-oxytocin injection, following additional oxytocin injection, and pre-discharge from the operating room, respectively (Fig. 1A). The degrees of shivering at the different time points are shown in Fig. 1B Both the highest incidence of shivering and the highest degree of shivering occurred after skin disinfection.

**Table 1:** Comparisons of characteristics between shivering and non-shivering groups

Characteristics	Shivering (*N* = 89)	Non-shivering (*N* = 123)	P
Age, years, M ± SD	35.7 ± 4.6	37.6 ± 4.1	0.002
BMI, kg/m^2^, M ± SD	27.1 ± 3.4	27.1 ± 2.6	0.994
Gestational age, week, M ± SD	38.8 ± 1.8	38.7 ± 1.5	0.580
Enter OR time, N (%)			0.010
0–8 o’clock,	8 (9%),	5 (4.1%),	
8–12 o’clock,	37 (41.6%),	66 (53.7%),	
12–18 o’clock,	33 (37.1%),	49 (39.8%),	
18–24 o’clock	11 (12.4%)	3 (2.4%)	
Anxiety, median (IQR)	1 (1, 1)	1 (1, 1)	0.006
Visual analog scale, N (%)			< 0.001
0–3	66 (74.2)	115 (93.5)	
4–6	10 (11.2)	8 (6.5)	
7–10	13 (14.6)	0 (0)	
Type of delivery, N (%)			< 0.001
Elective	28 (31.5)	87 (70.7)	
Emergency	61 (68.5)	36 (29.3)	
Transfer origin, N (%)			< 0.001
From delivery room	35 (39.3)	9 (7.3)	
From ward	54 (60.7)	114 (92.7)	
Labor analgesia, N (%)	28 (31.5)	7 (5.7))	< 0.001
Time from labor analgesia, hour, median (IQR)	0 (0, 4.25)	0 (0, 0)	< 0.001
Membrane rupture, N (%)	42 (47.2)	62 (16.3)	< 0.001
Time from membrane rupture, hour, median (IQR)	0 (0, 7)	0 (0, 0)	< 0.001
Dilation of cervix, centimeter, median (IQR)	0 (0, 2.75)	0 (0, 0)	< 0.001
Labor stage, N (%)			< 0.001
Not in labor	49 (55.1)	102 (82.9)	
Latent period	23 (25.8)	18 (14.6)	
Active period	13 (14.6)	2 (1.6)	
Second stage of labor	4 (4.5)	1 (0.8)	
Time of labor entry, hour, M ± SD	5.0 ± 8.8	1.2 ± 3.7	< 0.001
Operation indications, N (%)			< 0.001
Scarred uterus,	34 (38.6)	80 (65)	
Fetal distress,	15 (17)	7 (5.7)	
Labor stagnation	4 (4.5)	1 (0.8)	
Twins	4 (4.5)	11 (8.9)	
Malposition,	7 (8)	5 (4.1)	
Intrauterine infection	12 (13.6)	4 (3.3)	
PIH	2 (2.3)	2 (1.6)	
Others	10 (11.4)	13 (10.6)	
Sensory block level, M ± SD	5.1 ± 0.8	5.0 ± 0.8	0.630
Operation duration, minute, M ± SD	47.7 ± 14.8	47.6 ± 12.7	0.970
Blood loss, milliliter, M ± SD	223.6 ± 79.8	210.6 ± 42.1	0.163

*M ± SD* Mean ± standard deviation, *IQR* Interquartile range, *OR* Operating room

### Univariate analysis between shivering and various risk factors

Table 2 shows the univariate association between intraoperative shivering and various risk factors. Age, time of operating room entry, anxiety, VAS, type of delivery, transfer location, cervical dilation, analgesia, time of analgesia, membrane rupture, time of membrane rupture, labor stage, time of labor entry, and operation indications all had statistically significant associations with shivering.

**Table 2:** Risk factors associated with shivering during cesarean section

Risk factors	Univariate logistic regression		Multivariate logistic regression	
	Odds ratio (95% CI)	P	Odds ratio (95% CI)	P
Age	0.90 (0.84–0.96)	0.002		
Operating room entry time	1.28 (0.87–1.88)	0.021		
Anxiety	2.05 (1.24–3.38)	0.005	1.77 (1.01–3.12)	0.048
Visual analog scale	4.10 (2.01–8.36)	< 0.001		
Elective^a^ vs. emergency	5.27 (2.91–9.52)	< 0.001	2.90 (1.45–5.79)	0.003
From ward^a^ vs. delivery room	8.21 (3.29–18.29)	< 0.001	3.83 (1.52–9.66)	0.004
Dilation of cervix	1.59 (1.26–2.00)	< 0.001		
Labor analgesia	7.61 (3.14–18.42)	< 0.001		
Time of labor analgesia	1.28 (1.12–1.46)	< 0.001		
Rupture of membranes	4.60 (2.44–8.68)	< 0.001		
Time from membrane rupture	1.02 (0.99–1.04)	0.205		
Labor stage				
Not in labor^a^	2.81 (1.77–4.47)	< 0.001		
Latent period	2.66 (1.32–5.38)	0.007		
Active period	13.53 (2.94–62.31)	0.001		
Second stage of labor	8.33 (0.91–76.48)	0.061		
Time of labor entry	1.12 (1.05–1.20)	< 0.001		
Operation indications	1.14 (1.02–1.27)	0.019		

^a^control; *95% CI* 95% confidence interval

### Multivariate logistic regression analysis

Factors with *P* < 0.2 or with clinical significance were included in the multivariate logistic regression analysis, with shivering as the dependent variable (Table 2). The results showed that anxiety, type of delivery, and transfer location were statistically significantly associated with intraoperative shivering. Pregnant women who had anxiety, underwent emergency delivery, and were transferred from the delivery room to the operating room had higher odds ratios (1.77, 2.90, and 3.83, respectively) for intraoperative shivering.

### Risk factors associated with shivering at different time points

Since the incidence of shivering was not the same at different time points, we analyzed the risk factors associated with shivering at each time point. Multivariate logistic regression analyses showed that different risk factors were associated with shivering at the different time points (Table 3). Initial shivering in the operating room (pre-operation shivering) was strongly associated with labor analgesia (odds ratio 10.59). At the post-disinfection time point when the shivering reached its peak incidence, the risk factors associated with shivering were anxiety, type of delivery, and transfer origin. Pregnant women who had anxiety, underwent emergency delivery, and were transferred from the delivery room to the operating room had higher odds ratios (1.80, 2.44, and 3.42) for shivering after skin disinfection in the operating room.

**Table 3:** Multivariate logistic regression analyses on risk factors associated with shivering at different time points

Time points	Patients with shivering, N (%)	Risk factors	Odds ratio (95% CI)	*P*
Pre-operation	13 (6.1)	Labor analgesia	10.59 (3.22–34.83)	< 0.001
Post-anesthesia	45 (21.2)	VAS	3.86 (2.11–7.07)	< 0.001
		Ward^a^ vs. delivery room	3.17 (1.37–7.33)	0.007
Post-disinfection	63 (29.7)	Ward^a^ vs. delivery room	3.42 (1.45–8.09)	0.005
		Elective^a^ vs. emergency	2.44 (1.13–5.29)	0.023
		Anxiety	1.80 (1.02–3.18)	0.043
Post-delivery	56 (26.4)	Elective^a^ vs. emergency	4.05 (1.77–9.30)	0.001
		Ward^a^ vs. delivery room	3.36 (1.46–7.77)	0.005
Post-oxytocin	46 (21.7)	Elective^a^ vs. emergency	5.27 (1.86–14.96)	0.002
		Ward^a^ vs. delivery room	5.77 (2.36–14.07)	< 0.001
Additional oxytocin	33 (15.6)	VAS	2.19 (1.00–4.80)	0.049
		Elective^a^ vs. emergency	28.67 (3.50–234.67)	0.002
Pre-discharge from OR	32 (15.1)	Ward^a^ vs. delivery room	3.53 (1.55–8.02)	0.003

^a^control; *VAS* Visual analog scale, *OR* Operating room, *95% CI* 95% confidence interval

### Risk factors associated with shivering in pregnant women transferred from different locations to the operating room

Since our analysis showed that the transfer origin was a risk factor associated with shivering in the operating room, we evaluated the pregnant women separately depending on where they transferred to the operating room from (from the obstetric ward versus the delivery room) (Table 4). The results showed that emergency delivery was a risk factor for pregnant women transferred from the obstetric ward to the operating room. We did not identify any risk factors associated with shivering in pregnant women who were transferred from the delivery room to the operating room.

**Table 4:** Risk factors associated with shivering during cesarean section in patients transferred from different locations to operating room

Risk factors	Univariate logistic regression		Multivariate logistic regression	
	Odds ratio (95% CI)	*P*	Odds ratio (95% CI)	*P*
From ward (*N* = 168)				
Outdoor temperature	0.76 (0.55–1.04)	0.088		
Anxiety	1.85 (0.93–3.66)	0.078		
Enter OR visual analog scale	1.17 (0.93–1.46)	0.182		
Visual analog scale	2.10 (0.77–5.78)	0.149		
Elective^a^ vs. emergency	2.99 (1.51–5.95)	0.002	2.99 (1.51–5.95)	0.002
Rupture of membranes	1.87 (0.73–4.83)	0.195		
Operation indications	1.10 (0.97–1.24)	0.134		
From delivery room (*N* = 44)				
Anxiety	1.89 (0.72–4.95)	0.193		
Enter OR visual analog scale	1.39 (0.99–1.96)	0.056		
Visual analog scale	3.24 (0.86–12.22)	0.082		

^a^control, *OR* Operating room, *95% CI* 95% confidence interval

### Changes in body temperature at different time points in pregnant women with or without shivering

We measured the body temperature at different time points after the pregnant women entered the operating room. As shown in Fig. 1C, the body temperature gradually decreased until reaching its nadir after oxytocin injection and then slowly increased afterwards. The amplitude of fluctuation in the body temperature throughout the intraoperative period was < 0.2 °C. The body temperature in pregnant women with or without shivering showed parallel changes without statistically significant differences at each time point.

## Discussion

The overall incidence of shivering during the intraoperative period was 42% in parturients undergoing cesarean section, which was consistent with previous reports. Pregnant women who had anxiety, underwent emergency delivery, and were transferred from the delivery room to the operating room had higher odds of developing shivering during the cesarean section.

A previous study showed that almost all pregnant women could experience anxiety on the day of the cesarean section [21]. Anxiety and stress can have adverse impacts on pregnant women and the fetus, as well as on postoperative recovery and the length of hospital stay [22, 23]. A previous study reported that anxiety could contribute to shivering during cesarean sections [15]. Our present study further confirmed this result. By adjusting for various risk factors in a multivariate logistic regression model, our study showed that pregnant women with anxiety had about 1.77 times higher odds ratio to exhibit intraoperative shivering compared to pregnant women without anxiety. At the peak incidence of shivering during the intraoperative period, anxiety was still a significant risk factor contributing to shivering. This result suggested that healthcare providers should pay attention to the anxiety level of pregnant women. Efforts to minimize anxiety might decrease the incidence of shivering and improve the pregnancy outcomes.

Our results also showed that emergency delivery could increase the incidence of shivering compared to elective delivery. Emergency delivery was reported to be associated with several negative pregnancy outcomes, including post-traumatic stress disorder for the mother and fetal complications, such as low birth weight and increased morbidity and mortality [24]. Emergency cesarean section can also increase the anxiety level [25]. This suggested emergency cesarean section could at least partially affect anxiety and contribute to the development of shivering during the intraoperative period. A recent study showed that compared to elective cesarean section, emergency cesarean section was more likely to have adverse outcomes, such as postoperative wound infection, hemorrhage, urinary tract infection, and fever [26]. Our study showed that emergency cesarean section was also associated with a higher incidence of intraoperative shivering, which has never been reported previously. Physicians should take extra precautions for pregnant women who undergo emergency cesarean section.

In our hospital, pregnant women who enter labor are admitted to the delivery room for natural delivery. Therefore, parturients scheduled for cesarean section can be transferred from either the obstetric ward or the delivery room to the operating room. Here, we showed that pregnant women transferred from the delivery room to the operating room had a higher incidence of shivering than those transferred from the obstetric ward to the operating room, even after adjusting for the anxiety level, type of delivery, and other pregnancy-related factors. This may be because parturients transferred from the delivery room are all emergency cases. Other possible causes are that most parturients transferred from the delivery room had already received epidural labor analgesia and were more likely to have membrane rupture with increased pain intensity. Labor analgesia and membrane rupture can lead to heat loss, and increased pain can cause anxiety. All of these might affect the incidence of shivering. In addition, other factors, such as the environment or treatments in the delivery room, might be responsible for the increased incidence of shivering. Further studies are required to examine these factors to reduce the risk of intraoperative shivering.

Since the transfer origin was associated with the incidence of shivering, we assigned the pregnant women into either the obstetric ward group or the delivery room group for analysis. Our results only showed the type of delivery as a risk factor for shivering in women transferred from the obstetric ward to the operating room. We did not identify any risk factors for shivering in women transferred from the delivery room to the operating room. One potential explanation is that all pregnant women transferred from the delivery room received emergency surgery since they were already in labor. Our results indicated that emergency surgery contributed to shivering, which might mask subtle contributions from other risk factors in the women transferred from the delivery room. Another possible explanation is the small sample size in the delivery room group (168 in the obstetric ward group and 44 in the delivery room group), which lacked adequate statistical power to show the relationship between the risk factors and shivering.

We further calculated the incidence of shivering at different time points during the intraoperative period. The incidence and degree of shivering gradually increased once the pregnant women entered the operating room and reached the highest levels after skin disinfection. This might suggest that changes in the delivery location, anesthetic medication, and process of skin disinfection could have synergistic effects to induce shivering. The initial shivering in the operating room was strongly associated with the labor analgesia, suggesting that the analgesics used during cesarean sections might induce shivering. Previous studies have reported that ropivacaine and lidocaine could increase shivering during cesarean sections [27, 28]. Anesthesia may cause vasodilation, and cold disinfectant further promotes chills. However, the incidence of shivering decreased after delivery, although baby delivery induced further heat loss, possibly because the parturients experienced substantial anxiety relief after the baby was delivered. Furthermore, during all time points, only shivering post-disinfection was affected by anxiety (Table 3).

Shivering is closely related to the body temperature [3, 29]. Low body temperature has been reported as a risk factor for shivering in different surgical operations [30, 31]. Active warming was confirmed to effectively reduce the shivering incidence during cesarean sections [32]. In the present study, we measured the body temperature in pregnant women during the intraoperative period and further explored the relationship between the body temperature and shivering throughout the course of a cesarean section. The results showed that the body temperature initially decreased and then increased during the intraoperative period. The nadir of the body temperature occurred after oxytocin injection. Body temperature can be affected by various factors. In the initial intraoperative period, the body temperature drop could be caused by the combined effects from heat loss due to vasodilation in the anesthesia and the removal of warm amniotic fluid from the body. The application of cold disinfection solution could also lower the body temperature. In the later intraoperative period, oxytocin injection might be responsible for the rising temperature on the skin surface. Nevertheless, we speculated that the body temperature was not a risk factor for intraoperative shivering because 1) the time point for the lowest body temperature (post-oxytocin) was later than the time point for the peak incidence of shivering (post-disinfection); 2), the difference between the highest and lowest body temperatures was < 0.2 °C, which might have no clinical impact on the parturients; 3), the body temperature of parturients with or without shivering showed parallel changes without statistically significant differences at each time point.

The strengths of our study included the measurement of multiple risk factors, including membrane rupture, daytime versus nighttime surgery, and emergency versus elective cesarean section, which have never been studied previously. We also measured the incidence and degree of shivering at different time points throughout the course of the cesarean section. We constructed several multivariate logistic regression models to comprehensively evaluate these risk factors for shivering. The limitations of the present study are the single-center research design with a small sample size in subgroup analyses. Anxiety was determined based on the patient’s self-reported symptoms but was not systematically measured. This could introduce bias in our results.

## Conclusions

In summary, our present study showed that anxiety, emergency delivery, and transfer from the delivery room to the operating room could increase the risk of shivering development in pregnant women during cesarean sections. The peak incidence of shivering occurred after skin disinfection in the operating room. Further investigations are warranted to guide interventional therapy and minimize these risk factors to decrease the risk of shivering.

## Data Availability

The raw data supporting the conclusions of this article will be made available by the corresponding author, without undue reservation.

## Abbreviations

ASA: American Society of Anesthesiologists
VAS: Visual Analogue Scale
